# Molecular Genetic Analysis of Ukrainian Families with Congenital Cataracts

**DOI:** 10.3390/children10010051

**Published:** 2022-12-26

**Authors:** Xiaodong Jiao, Mariia Viswanathan, Nadiia Fedorivna Bobrova, Tatiana Viktorivna Romanova, J. Fielding Hejtmancik

**Affiliations:** 1 Ophthalmic Genetics and Visual Function Branch, National Eye Institute, National Institutes of Health, Bethesda, MD 20892, USA; 2 Vision Care Readiness Section, Vision Center of Excellence, Defense Health Agency Research and Engineering, Bethesda, MD 20889, USA; 3 Department of Pediatric Ophthalmic Pathology, State Institution “The Filatov Institute of Eye Diseases and Tissue Therapy of The National Academy of Medical Sciences of Odessa, Ukraine”, 65000 Odessa, Ukraine

**Keywords:** lens, autosomal dominant congenital cataract, whole-exome sequencing

## Abstract

This study was designed to identify the pathogenic variants in five Ukrainian families with autosomal dominant congenital cataracts. Cataracts can be defined broadly as any opacity of the crystalline lens. Lens development is orchestrated by transcription factors. Disease-causing variants in transcription factors and their developmental target genes, including the lens crystallins, are associated with congenital cataracts and other eye diseases. Whole-exome sequencing identified heterozygous disease-causing variants in five Ukrainian families with autosomal dominant congenital cataracts and cosegregation with cataracts was confirmed using Sanger sequencing. Family 97001 showed a missense variant (c.341T>A: p.L114Q) in HSF4; family 97003 showed a missense variant (c.53A>T: p.N18I) in CRYGA; family 97004 showed a missense variant (c. 82G>A: p.V28M) in GJA3; family 97006 showed a missense variant (c.83C>T: p. P28L) in CRYGC; and family 97008 showed a single-base insertion resulting in a frameshift (c.443_444insA: p. Met148IfsTer51) in PAX6. All five families are associated with congenital cataracts. Overall, we report four novel mutations in HSF4, CRYGA, CRYGC and PAX6, and one previously reported mutation in GJA3 that cause autosomal dominant congenital cataracts.

## 1. Introduction

Cataracts can be defined as an opacification of the crystalline lens that interferes with the transmission and focusing of visual images on the retina. Opacification occurs when the refractive index of the lens varies significantly over distances roughly approximating the wavelength of the transmitted light [1,2]. Cataracts as lens opacities have been recognized as a group of well-known diseases for millennia [3,4]. Congenital cataract is a significant cause of vision loss worldwide. While congenital cataracts are much less common than age-related cataracts, they can lead to permanent blindness by interfering with the sharp focus of light on the retina, resulting in failure to establish appropriate visual cortical synaptic connections with the retina [5]. Estimates of the incidence of congenital cataracts vary from 12 to 136 per 100,000 births, with between 8.3 and 25 percent of congenital or infantile cataracts being hereditary, varying in different populations [6,7,8,9]. The cataract prevalence among Ukrainian children in some regions reaches up to 6.1 per 10,000, which is above the average compared to other countries in Europe [10], with cataract prevalence rates among children in European countries being 2.49–3.46 per 10,000 children in the UK, 4.0 per 10,000 children in Sweden, and 2.3 per 10,000 in Denmark [11]. Genetic studies have identified mutations in over 55 causative genes for congenital or other early-onset forms of cataract [12]. Approximately 33%, 26%, and 18% of the total mutations causing isolated congenital cataract have been reported in genes encoding crystallin, growth factors, and connexin proteins, respectively [13]. In this study, we have undertaken whole-exome sequencing (WES) in order to identify pathogenic variants underlying autosomal dominant congenital cataracts in five Ukrainian families.

## 2. Methods

### 2.1. Study Design

The aim of this study is to characterize the genes and mutations associated with congenital and hereditary cataracts in Ukrainian families. Individual patients with cataracts were ascertained at the Filatov Institute of Eye Diseases and Tissue Therapy of the Academy of Medical Sciences of Ukraine; pedigrees were drawn and additional family members, if any, were recruited. DNA was isolated and sent to the OMGS/OGVFB/NEI/NIH for sequencing and analysis.

### 2.2. Clinical Analysis

Families were recruited at the Pediatric Department of the Filatov Institute of Eye Diseases and Tissue Therapy of the Academy of Medical Sciences of Ukraine. The study protocols adhered to the Tenets of the Declaration of Helsinki and were approved by the National Eye Institute IRB on 4 November 2019 (Bethesda, MD, USA 16-EI-0104, last review 2022) and The Filatov Institute of Eye Diseases and Tissue Therapy of The National Academy of Medical Sciences of Ukraine IRB (Odesa, Ukraine, 1, last review 2021). All the family members participating in this study gave written informed consent and underwent ophthalmic examination, which included tests of visual function such as visual acuity, refraction, IOP, slit lamp and fundus examination. Detailed family and medical histories were compiled by reviewing available medical records from all the affected individuals and their relatives from the five families diagnosed with autosomal dominant inherited congenital cataract. Genomic DNA was extracted from white blood cells using a blood and cell culture DNA kit (QIAGEN, Manchester, UK). DNA concentrations were estimated using a NanoDrop (Thermo Scientific, Carlsbad, CA, USA).

### 2.3. Whole-Exome Sequencing and Bioinformatics Analysis

DNA samples from a single affected individual from each family were subject to whole-exome sequencing (Novogen, Sacramento, CA, USA). If no causative mutation was definitively identified in a family, DNA from a second individual was sequenced. Paired-end sequencing was performed on a NovaSeq PE 150, and the short-read sequence data were aligned to the GRCh38 human reference sequence. To visualize genomic data from WES results, we used the Integrative Genome Viewer Browser (https://igv.org, accessed on 1 November 2022). Variants were filtered using 1000 Genomes and Genome Aggregation Database (gnmAD) project Minor Allele Frequency (MAF) <0.005 and in silico pathogenicity prediction tools SIFT (http://sift.jcvi.org, accessed on 1 November 2022), Polyphen2 (http://genetics.bwh.harvard.edu/pph2, accessed on 1 November 2022), Mutation Taster (http://mutationtaster.org, accessed on 1 November 2022), LRT (http//genetics.wustl.edu/jflab/lrt_query.html, accessed on 1 November 2022) and Provean (http://jcvi.org/research/provean, accessed on 1 November 2022). The presence of the variant was verified using Sanger sequencing.

### 2.4. Sanger Sequencing

Sanger sequencing was performed to validate variants identified by whole-exome sequencing and confirm cosegregation of the variant with cataracts. Specific primer pairs were designed using Primer3 (http://primer3.ut.ee/, accessed on 1 November 2022) (Table S1). The amplicons were sequenced on an ABI 3500 Genetic Analyzer (Applied Biosystems, Foster City, CA, USA) and analyzed with Seqman version 5.1 (DNAStar Lasergene 8; Madison, WI, USA) and Mutation Surveyor (SoftGenetics, State College, PA, USA) software.

## 3. Results

**Figure 1:**
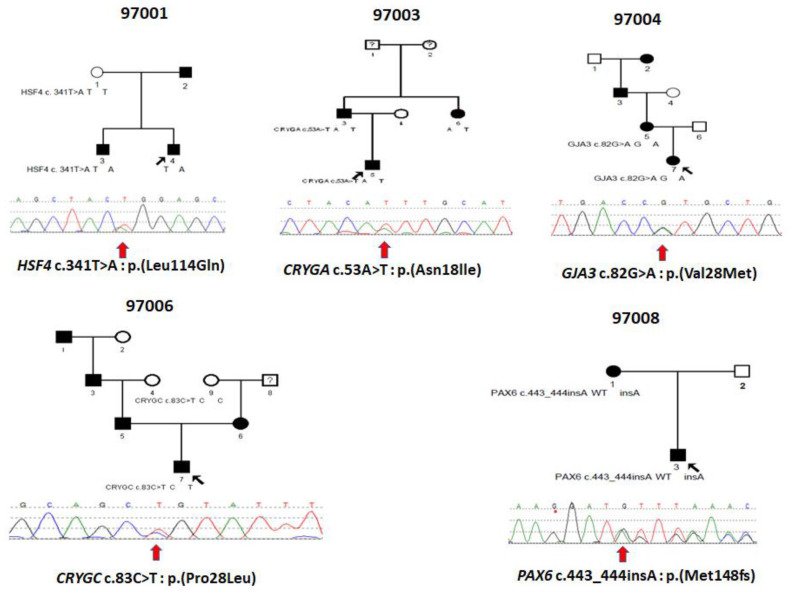
Pedigrees of Ukrainian families 97001, 97003, 97004, 97006 and 97008 with inherited congenital cataracts and sequence chromatograms of variants in the associated genes showing in the probands of each of the five families. Circles represent females and squares males. Filled symbols indicate individuals affected by congenital cataracts. Black arrows indicate probands and red arrows below the sequence tracings indicate the sequence variations.

Five Ukrainian families with autosomal dominant congenital cataracts (97001, 97003, 97004, 97006, and 97008) were recruited from the pediatric department of the Filatov Institute of Eye Diseases and Tissue Therapy of the Academy of Medical Sciences of Ukraine (Figure 1). The patients’ available medical records confirmed that all affected individuals showed isolated congenital or early childhood cataracts without any non-ocular anomalies (Table 1).

### 3.1. Family 97001

**Figure 2:**
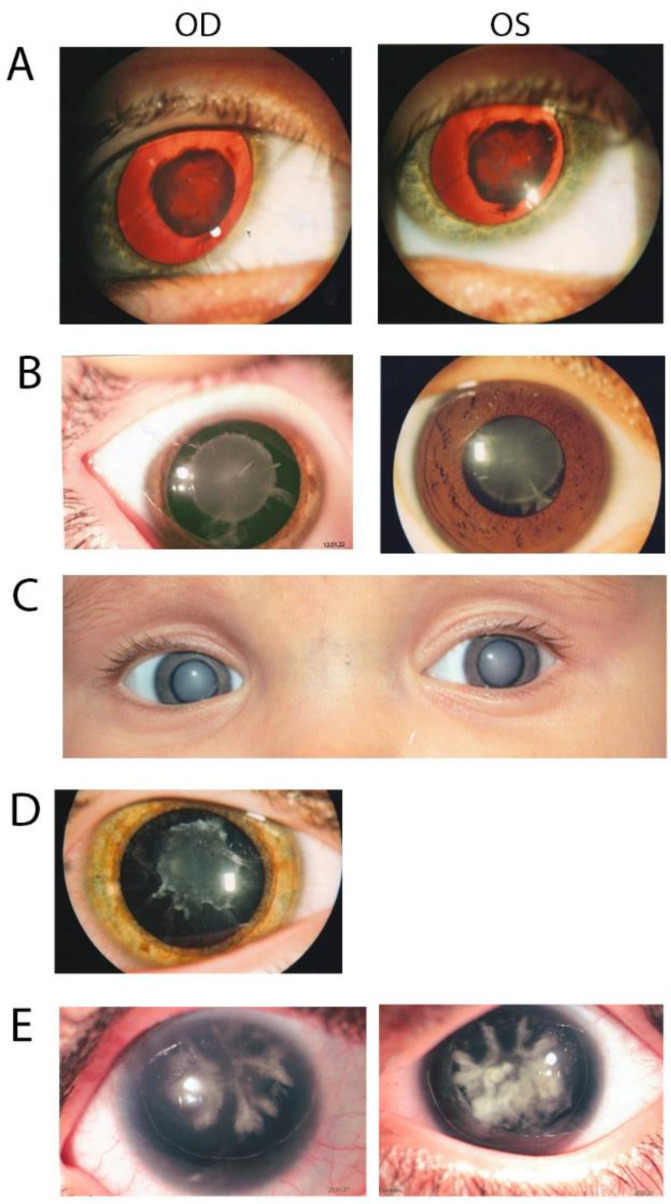
Photographs showing eyes of affected patients from the five families. (**A**) Red reflex image of proband 4 of family 97001 with nuclear cataract. (**B**) Image of proband 5 of family 97003 showing a congenital nuclear cataract with cortical riders. (**C**). Eye picture of proband 7 of family 97004 with congenital nuclear cataract. (**D**) Image of proband 7 of family 97006 showing congenital nuclear cataract. (**E**) Image of proband 3 of family 97008 with a coralliform congenital cataract.

**Figure 3:**
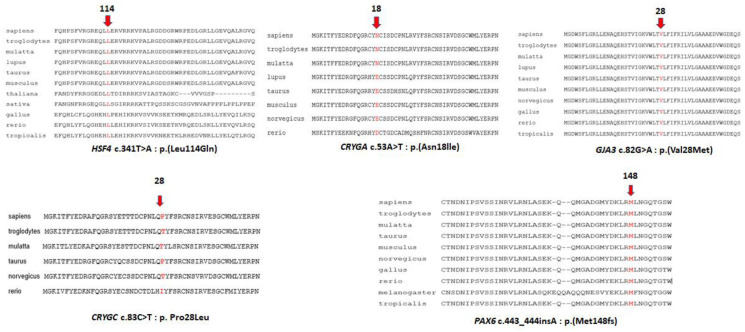
Multiple sequence alignments from different species of regions surrounding cataract mutations. Arrows and the amino acids in red indicate the position of the mutation.

**Figure 4:**
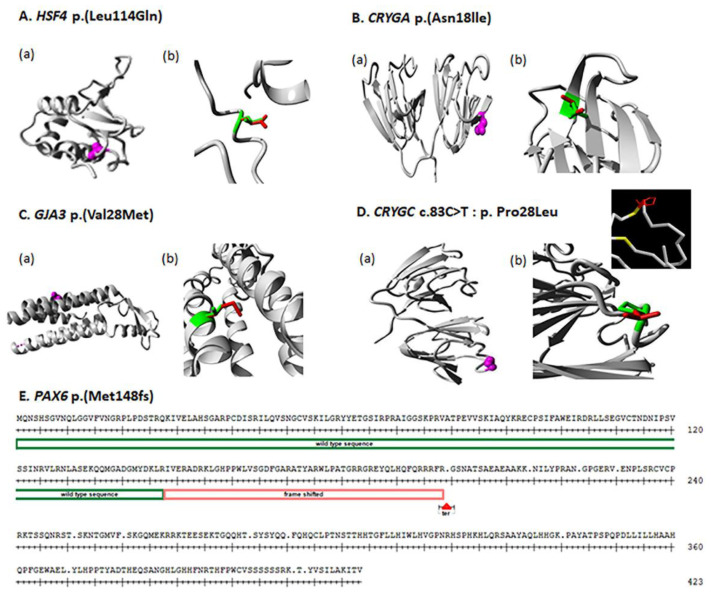
(**A**–**D**) Protein structure analysis for all detected variants by HOPE software. (**A**–**D**) (**a**): Each variant shows the position of amino acid in protein structure; the mutated residue is colored magenta. (**A**–**D**) (**b**): For each identified variant, enlarged views of both the wild-type and the mutant residue are shown and colored green and red, respectively. (**E**) Protein sequence of PAX6 gene, the red box indicates where the position of mutation started, resulting in a frame shift.

This is a two-generation family with autosomal dominant congenital hereditary cataracts. All three affected individuals have nuclear cataracts in both eyes, and individuals 3 and 4 had surgery carried out on both eyes. Affected individual 4 (Figure 2A) showed a novel heterozygous variant NM_001040667 c.341T>A: p. Leu114Gln in exon 5 of HSF4 (Figure 3 and Figure 4A), and cosegregation with cataracts was confirmed by Sanger sequencing of affected individuals 3 and 4 and the unaffected mother (Figure 1). Amino acid residue Leu114 is completely conserved among species ranging from humans to zebrafish (Figure 3), suggesting that it is critical for HSF4 function. The significance of the variation was predicted using PolyPhen-2, Mutation Taster, SIFT and Provean, and all five programs suggested that it was pathogenic (Table 2). This variation was not found in the gnomAD database. Considering the low allele frequency in Europeans and the known role of *HSAF4* mutations in cataractogenesis, this places the mutation in the PM2 category of the ACMG guidelines.

### 3.2. Family 97003

In this family with a clear diagnosis of autosomal dominant congenital cataracts, the proband, individual 5, was diagnosed at 3 years of age with congenital nuclear cataract (Figure 2B). The boy underwent surgery at 5 years old, and affected individual 6 underwent surgery at 24 years old on both eyes. Affected individual 3, who underwent WES, also underwent surgery on his left eye at 28 years old. After genetic variant analysis and filtering, a novel heterozygous variant NM_014617 c.53A>T: p. Asn18lle in exon2 of CRYGA was found. This change has an allele frequency of 0.005 (rs61743752) in Europeans and was predicted to be causative in three of the five predictive programs. Asn18 is conserved among primates, but is substituted by glutamic acid in other mammals and aspartic acid in fish (Figure 3). Cosegregation with cataracts was confirmed by direct sequencing (Figure 1).

### 3.3. Family 97004

Two samples were available from affected individuals in this four-generation pedigree. Both individuals were diagnosed with congenital cataracts, patient 7 at two months and patient 5 at seven years, and both underwent cataract surgery. A DNA sample from affected individual 5 was sent for WES, and variant annotation and filtering showed a heterozygous variant NM_021954 c. 82G>A: p. Val28Met in exon2 of GJA3 and was not found in the gnomAD database. Affected individual 7, who is a daughter of individual 5, also carries the same mutation. This variant is predicted to be pathogenic by all five predictive programs and has previously been reported as a pathogenic variant (Table 2).

### 3.4. Family 97006

Individual 7 from family 97006 was diagnosed with a congenital hereditary nuclear cataract (Figure 2D) at 1 month of age and underwent surgery on his right eye a 1 years old and on his left eye at 2 years old. WES was carried out on this individual, and after variant analysis and filtering, a heterozygous variant in *CRYGC*, NM_020989 c.83C>T: p. (Pro28Leu) in exon2 of *CRYGC* was identified (Figure 1, Table 2). It was not found in the gnomAD database. Unaffected Individual 9 has a normal allele at this position, suggesting that individual 8, who was not examined and from whom no DNA sample is available, might carry a cataractogenic variation inherited by individual 6 and possibly 7. Because DNA samples are not available from individuals 1, 3, 5, or 6, it is not possible to determine whether individual 7 inherited the causative variant from the maternal or paternal lines, or both. While only PROVEAN of the five prediction programs used indicated pathogenicity, this change is included as possibly causative because of its low allele frequency and its position within and immediately adjacent to one of the tyrosines forming the non-Greek key aromatic pair, Y17–Y29, in the first Greek key motif (Figure 4D, insert).

### 3.5. Family 97008

In family 97008, a mother–son pair were diagnosed with congenital cataracts and aniridia at birth. In addition, the affected mother had additional eye pathologies including corneal degeneration, secondary glaucoma, partial optic atrophy, nystagmus and partial ptosis. She had cataract surgery at 5 years of age. Her affected son also showed other eye pathologies including secondary glaucoma in the stage of decompensation in addition to cataracts (Figure 2E). No surgery had been performed on him prior to exam (Table 1). WES of a DNA sample from individual 3 disclosed a novel heterozygous single-base insertion NM_001310159 c.443_444 insA, p. Met148IfsTer51, in PAX6, resulting in a frameshift mutation with premature termination 51 amino acids downstream. This variant showed in both affected individuals of family 97008 (Figure 1), and was not found in the gnomAD database.

## 4. Discussion

Here, we describe five heterozygous disease-causing variants in five Ukrainian families with autosomal dominant congenital cataracts. Of these, four are novel and one has been reported previously as causing congenital-cataracts-associated genes. Mutations in all the genes have been associated with congenital cataracts, and the genes themselves are known to be important in lens development, homeostasis, and transparency.

### 4.1. HSF4

Mutations in the human *HSF4* gene have been reported in both autosomal dominant and recessive cataracts, as well as having been associated with age-related cataracts [12]. To date, twenty-seven mutations of HSF4 gene have been reported to cause cataracts [15,16,17,18,19,20,21,22,23,24,25,26,27,28,29,30,31,32,33]. In this study, a novel missense variant c.341T>A: p. Leu114Gln was found to cosegregate with the disease phenotype in family 97001 (Figure 1). The Leu114 residue is conserved among different species (Figure 3), suggesting that it is essential for protein function. the wild-type and mutant amino acids differ in size. The mutant residue is bigger than the wild-type residue. The residue is located in a small helical region in a loop on the surface of the protein; mutation of this residue can disturb interactions with other molecules or other parts of the protein. As leucine is hydrophobic and glutamine is polar, the mutation might cause loss of hydrophobic interactions with other molecules on or near the surface of the protein (Figure 4).

### 4.2. CRYGA

Cataracts in family 97003 are associated with a novel heterozygous variant, c.53A>T; p. Asn18lle, of CRYGA responsible for an autosomal dominant congenital cataract (Figure 1). Perhaps because CRYGA is not as highly expressed as CRYGC or CRYGD in the human lens, only one splice and two missense mutations in CRYGA have been reported in previous studies [18,34,35], both families with autosomal dominant congenital cataract. The wild-type and mutant amino acids differ in size, the mutant residue is smaller than the wild-type residue, and this will cause a possible loss of external interactions. The hydrophobicities of the wild-type and mutant residues differ, and this difference in hydrophobicity is predicted to affect hydrogen bond formation with the water shell of the protein and thus its stability [36] (Figure 4).

### 4.3. GJA3

In humans, GJA3 has been associated with a variety of inherited forms of cataract, the most common of which is nuclear or lamellar autosomal dominant congenital cataract [13], but also including autosomal recessive and age-related cataract [12]. Family 97004 presented with a previously reported heterozygous missense variant c.82G>A; p. Val28Met of GJA3 gene [14] (Figure 1, Table 2). This mutant residue is highly conserved from humans to Xenopus (Figure 3), and the mutant residue, which resides in a transmembrane domain α-helix, is larger than the wild-type residue, which could cause crowding with the adjacent α-helix or possibly disturb contacts with other transmembrane domains or with the lipid-membrane itself (Figure 4).

### 4.4. CRYGC

In humans, 35 separate mutations in CRYGC have been associated with cataracts, all of which are nuclear or lamellar autosomal dominant or sporadic congenital cataracts http://cat-map.wustl.edu (accessed on 1 November 2022) [12,13]. The status of the p. (Pro28Leu) change is uncertain because of the weak support from the predictive programs examined. However, the position of this change inside 1 of 14 aromatic pairs, including Tyrosine corners stabilizing the Greek key motifs of the CRYGC protein, suggests it might be deleterious. As these aromatic pairs contribute significantly to the stability of the γ-crystallins [37,38], it is likely that the close proximity of the Pro28Leu change decreases the pi-stacking interaction of Y17 and Y29, thus destabilizing the CRYGC protein. Similarly, while the proline residue at position 28 is only conserved among primates and bovines, it does allow torsional angles that are excluded for most other amino acids except glycine, providing some theoretical support for this variant possibly being causative.

### 4.5. PAX6

In humans there are about 500 mutations reported in the *PAX6* gene ([12], http://cat-map.wustl.edu, accessed on 1 November 2022), (https://www.lovd.nl/, accessed on 1 November 2022). In this study, a mother–son pair in family 97008 showed a combination of congenital cataract, aniridia, secondary glaucoma, and nystagmus (Table 1). Aniridia is a developmental anomaly of the eye in which a variable degree of hypoplasia or absence of iris is often associated with other ocular features, including cataract, glaucoma and corneal opacification and vascularization secondary to limbal stem cell deficiency. Aniridia is commonly seen with *PAX6* mutations. Affected individuals in family 97008 showed heterozygous single-base insertion (NM_001310159 c.443_444 insA: p. Met148IfsTer51) in *PAX6*, resulting in a frameshift mutation (Figure 1, Table 2). Met148 is very conserved, being located in the paired domain that is important for DNA binding. The single base insertion creates a frameshift followed by 51 random amino acids and then is predicted to create a premature termination site in exon 4, which should result in nonsense mediated decay, or eliminate the C-terminal part of the paired domain as well as all of the homeobox domain and transactivating domain of any transcript that escapes nonsense mediated decay, which would be predicted to completely inactivate the PAX6 protein (Figure 4).

Some, if not most, congenital cataracts are caused by mutations that severely destabilize crystallins or disrupt lens homeostasis, often through inducing cellular stress and hence activation of the unfolded protein response and subsequent apoptosis. Lens development follows a well-documented timed sequence. The location of a lens opacity provides information about the time at which the pathologic process intervened, and cataracts are no exception. The different genes identified in these families probably act through separate pathways: aberrant development in the cases of PAX6 and HSF4, disrupted intercellular signaling for GJA3, and instability of major lens structural proteins in the case of CRYGA and CRYGC. However, they reach a final common pathway. Mutations in crystallins or other lens proteins sufficient in and of themselves to damage the cells through disrupted development, homeostasis or protein aggregation usually damage lens cells directly, in some cases invoking the unfolded protein response and/or apoptosis, with resulting congenital cataracts [39]. Of the genes involved in these families, only PAX6 has major effects outside the lens, and the cataracts in those cases are often accompanied by additional ocular defects and, thus, seem likely to be secondary to the developmental problems in the broader eye field resulting from loss of this protein. Like most Mendelian cataracts, those in this group of families are inherited as autosomal dominant traits. One additional observation is that while the number of Ukrainian cataract families recruited to date is small, they all have autosomal dominant cataracts, in general agreement with other studies of European populations [9,40,41,42]. In contrast, in Pakistan, the majority of inherited cataracts show an autosomal recessive pattern [28].

## 5. Conclusions

Here, we report four novel and a previously reported variants in five Ukrainian families, with one in *CRYGC* only being probably causative, all in genes that are associated with inherited congenital cataracts. These results expand the mutation spectrum of congenital cataracts and should be helpful clinically for the genetic diagnosis of congenital cataracts. In addition, identification of mutations responsible for autosomal dominant congenital cataracts in this study further highlights the significant genetic contribution in familial patients of Ukrainian descent.

## Figures and Tables

**Table 1:** Clinical data for the Congenital Cataract patients.

Patient	Sex	Age at Diagnosed	Clinical Diagnosis	Other Eye Pathologies	Visual Acuity (OD/OS)	Surgery
97001-3	M	6 y.o.	Congenital nuclear cataract	amblyopia, concomitant strabismus	0.7/0.5	Yes
97001-4	M	3 y.o.	Congenital hereditary nuclear cataract	axial myopia, amblyopia, concomitant strabismus, lower eyelid tum(Entropon)	0.7/0.5	Yes
97003-3	M	5 y.o.	Congenital cataract, nuclear pulverulent with sutural component and cortical riders	OD:High-grade myopia, complex myopic astigmatism; OS:Myopia is mild, myopic astigmatism	0.02/0.4	Yes
97003-5	M	3 y.o.	Congenital hereditary cataract		0.4/0.015	Yes
97003-6	F	6 y.o.	Congenital cataract	OS:Medium-grade hypermetropia, Hypermetropic astgmatism	1.0/0.3	Yes
97004-5	F	7 y.o.	Congenital cataract		0.85/0.85	Yes
97004-7	F	2 month	Congenital cataract, total		0.1/light perception	Yes
97006-7	M	1 month	Congenital hereditary nuclear cataract	Microphthalmos, concomitant strabismus, nystagmus	0.2/0.4	Yes
97008-1	F	At the birth	Congenital cataract	congenital aniridia corneal degeneration;secondary glaucoma, partial optic atrophy; nystagmuscongenital aniridia	Can’t establish/Hm + 10.0 D	Yes
97008-3	M	At the birth	Congenital cataract, corraliform	congenital aniridia, secondary glaucoma, nystagmus	0.12/0.1	No

**Table 2:** Mutations Identified In Families With Congenital Cataract.

Fam	Gene	Nuc Change	AA Change	PP2	Pro	MT	LRT	Sift	Type	ACMG	Note
97001	*HSF4*	c.341T>A	p.(Leu114Gln)	D	Del	DC	D	D	Het	PM2	New
97003	*CRYGA*	c.53A>T	p.(Asn18lle)	B	Del	N	D	D	Het	PM2	New
97004	*GJA3*	c.82G>A	p.(Val28Met)	D	Del	DC	D	D	Het	PS1	Ref [14]
97006	*CRYGC*	c.83C>T	p.(Pro28Leu)	B	Del	N	N	T	Het	PM2	New
97008	*PAX6*	c.443_444insA	p.(Met148fs)	N/A	N/A	N/A	N/A	N/A	Het	PVS1	New

PP2: polyphen 2, Pro: Provean, MT: Mutation Taster, Type, D: damaging, B: benign, N:neutral; Del: deleterious, DC: disease causing, T: tolerated, Het: heterozygous, New: novel; ACMG: ACMG category.

## Data Availability

All relevant data are included in the manuscript.

## Supplementary Materials

The following supporting information can be downloaded at: https://www.mdpi.com/article/10.3390/children10010051/s1, Table S1: List of Primers used in this study.
