# Supplementary material for: Molecular Genetic Analysis of Ukrainian Families with Congenital Cataracts
Source: Children (Basel). 2022 Dec 26;10(1):51. doi: 10.3390/children10010051 (PMC9856374; doi:10.3390/children10010051)
Supplement: Supplementary file 1 [file children-10-00051-s001.zip › children-2094984-supplementary.pdf]

children

MDPI

Table S1. List of Primers used in this study.

Name	Sequence
HSF4 c.341T>A p.(Leu114Gln)	5′AgACGGTTTTCGGAAGGTG TCGCCCATAAGTCTAGATGGA3′
CRYGA c.53A>T p.(Asn18lle)	5′TCTGTGGTGTGTGGGGATG ATTCGGGGTACTTGCCTC3′
GJA3 c.82G>A p.(Val28Met)	5′CCATCCCAGTACCATCCAG GCGTGGACACGAAGATGAT3′
CRYGC c.83C>T p .(Pro28Leu)	5′GCCTGTTAGGAGCAAATAATGT CCATCCATTGCTGGTATTCG3′
PAX6 c.443_444insA p.(Met148fs)	5′GGTTGTGGGTGAGCTGAGAT AAGCCCTGAGAGGAAATGGT3′
